# Comparing GPT-4 and Human Researchers in Health Care Data Analysis: Qualitative Description Study

**DOI:** 10.2196/56500

**Published:** 2024-08-21

**Authors:** Kevin Danis Li, Adrian M Fernandez, Rachel Schwartz, Natalie Rios, Marvin Nathaniel Carlisle, Gregory M Amend, Hiren V Patel, Benjamin N Breyer

**Affiliations:** 1 Department of Urology University of California San Francisco San Francisco, CA United States; 2 Department of Epidemiology and Biostatistics University of California San Francisco San Francisco, CA United States; 3 Department of Anesthesia and Perioperative Care University of California San Francisco San Francisco, CA United States; 4 Division of General Internal Medicine, Department of Medicine University of California San Francisco San Francisco, CA United States; 5 Department of Urology Icahn School of Medicine at Mount Sinai New York, NY United States

**Keywords:** artificial intelligence, ChatGPT, large language models, qualitative analysis, content analysis, buried penis, qualitative interviews, qualitative description, urology

## Abstract

**Background:**

Large language models including GPT-4 (OpenAI) have opened new avenues in health care and qualitative research. Traditional qualitative methods are time-consuming and require expertise to capture nuance. Although large language models have demonstrated enhanced contextual understanding and inferencing compared with traditional natural language processing, their performance in qualitative analysis versus that of humans remains unexplored.

**Objective:**

We evaluated the effectiveness of GPT-4 versus human researchers in qualitative analysis of interviews with patients with adult-acquired buried penis (AABP).

**Methods:**

Qualitative data were obtained from semistructured interviews with 20 patients with AABP. Human analysis involved a structured 3-stage process—initial observations, line-by-line coding, and consensus discussions to refine themes. In contrast, artificial intelligence (AI) analysis with GPT-4 underwent two phases: (1) a naïve phase, where GPT-4 outputs were independently evaluated by a blinded reviewer to identify themes and subthemes and (2) a comparison phase, where AI-generated themes were compared with human-identified themes to assess agreement. We used a general qualitative description approach.

**Results:**

The study population (N=20) comprised predominantly White (17/20, 85%), married (12/20, 60%), heterosexual (19/20, 95%) men, with a mean age of 58.8 years and BMI of 41.1 kg/m^2^. Human qualitative analysis identified “urinary issues” in 95% (19/20) and GPT-4 in 75% (15/20) of interviews, with the subtheme “spray or stream” noted in 60% (12/20) and 35% (7/20), respectively. “Sexual issues” were prominent (19/20, 95% humans vs 16/20, 80% GPT-4), although humans identified a wider range of subthemes, including “pain with sex or masturbation” (7/20, 35%) and “difficulty with sex or masturbation” (4/20, 20%). Both analyses similarly highlighted “mental health issues” (11/20, 55%, both), although humans coded “depression” more frequently (10/20, 50% humans vs 4/20, 20% GPT-4). Humans frequently cited “issues using public restrooms” (12/20, 60%) as impacting social life, whereas GPT-4 emphasized “struggles with romantic relationships” (9/20, 45%). “Hygiene issues” were consistently recognized (14/20, 70% humans vs 13/20, 65% GPT-4). Humans uniquely identified “contributing factors” as a theme in all interviews. There was moderate agreement between human and GPT-4 coding (κ=0.401). Reliability assessments of GPT-4’s analyses showed consistent coding for themes including “body image struggles,” “chronic pain” (10/10, 100%), and “depression” (9/10, 90%). Other themes like “motivation for surgery” and “weight challenges” were reliably coded (8/10, 80%), while less frequent themes were variably identified across multiple iterations.

**Conclusions:**

Large language models including GPT-4 can effectively identify key themes in analyzing qualitative health care data, showing moderate agreement with human analysis. While human analysis provided a richer diversity of subthemes, the consistency of AI suggests its use as a complementary tool in qualitative research. With AI rapidly advancing, future studies should iterate analyses and circumvent token limitations by segmenting data, furthering the breadth and depth of large language model–driven qualitative analyses.

## Introduction

Recent advancements in artificial intelligence (AI), particularly in large language models, have significantly expanded their applications in health care and academic research. These developments raise critical questions about their potential and ethical use [1-3]. GPT-4, developed by OpenAI, is a large language model that uses deep learning algorithms, specifically the GPT, to process and generate human-like text [4]. Its training on diverse internet text sources through unsupervised learning enables it to interpret complex language data, making it a potentially invaluable tool for qualitative research [5]. This is especially important in areas where traditional qualitative data analysis is labor-intensive and requires expertise to understand subtle nuances [6]. Furthermore, it is unknown how AI-driven qualitative analysis may differ from human-driven analysis in research contexts.

Despite its potential, the application of AI and large language models to qualitative data remains underexplored [7,8]. Previous studies in the realm of qualitative data analysis have used traditional natural language processing (NLP) models, which often require benchmark-specific training and hand engineering, leading to a more constrained contextual understanding and inferencing abilities. For example, Lennon et al [9] combined human coding with an NLP system trained on internal data, significantly reducing coding time, while Cheligeer et al [10] used a model based on BERT (Bidirectional Encoder Representations from Transformers; Google) for faster keyword analysis. However, such models fall short of the advanced contextual and inferencing abilities exhibited by widely trained large language models like GPT-4, which has been shown to outperform traditional systems on standard NLP benchmarks [11]. Although the field is rapidly evolving, there remains a limited number of studies that directly compare AI-driven qualitative analysis with human-driven approaches [12-17].

In this study, we used GPT-4 to re-examine qualitative data from a previously published study of 20 patients with adult-acquired buried penis (AABP), a urological condition with significant psychosocial consequences, and compare its performance with that of human researchers [18]. Evaluating GPT-4 for qualitative analysis in this patient population is particularly important due to the unique and profound psychosocial distress associated with AABP, including issues related to body image, sexual function, and mental health. Understanding patients’ experiences through qualitative analysis can provide an increased understanding of their lived experiences. To accomplish these objectives, we created a series of generalizable prompts that allow the application of GPT-4 to qualitative analysis without requiring specialized knowledge or skills [19]. Finally, we evaluated the validity of our approach by measuring agreement between GPT-4 and human analysis and reliability by assessing if prompts consistently elicited similar outputs from the same data.

## Methods

### Data Source

Qualitative data were from a convenience sample of 20 patients who presented to urology clinics participating in TURNS (Trauma and Urologic Reconstructive Network of Surgeons), a multi-institutional collaborative research group focused on urologic trauma and reconstruction [18]. We conducted semistructured interviews focusing on the impact of AABP on personal relationships, social life, mental health, and physical health. Participants were interviewed for 15 to 30 minutes, and audio was transcribed electronically using Otter transcription software [20]. Interviews were conducted over Zoom live video conferencing [21]. For both human and GPT-4 qualitative analyses, only deidentified text transcripts were used, ensuring that the qualitative data were interpreted solely from text, providing a comparable basis for both human and AI-driven analyses.

### Human Analysis

Our human-driven analysis used a general qualitative description approach which differs from other qualitative methods in that the analytic process stays close to the data, describing informants’ experiences using their own language [22-24]. The research team initially reviewed interview transcripts, taking notes to capture observations and ideas and facilitate a comprehensive understanding of the overall content. This preparatory work informed the subsequent structured coding process. To ensure consistency and reliability, the team convened at three key stages, which were (1) before coding, to share initial text impressions and establish a standardized coding protocol; (2) after initiating line-by-line coding, to discuss applied codes and refine categorization strategies; and (3) to assess coder interrater reliability using weighted Fleiss κ coefficients [25]. Codes with a κ value below 0.75 were discussed among all authors until a coding consensus was reached. This approach enabled the identification and categorization of relevant subthemes and themes.

### AI Analysis

**Figure 1:**
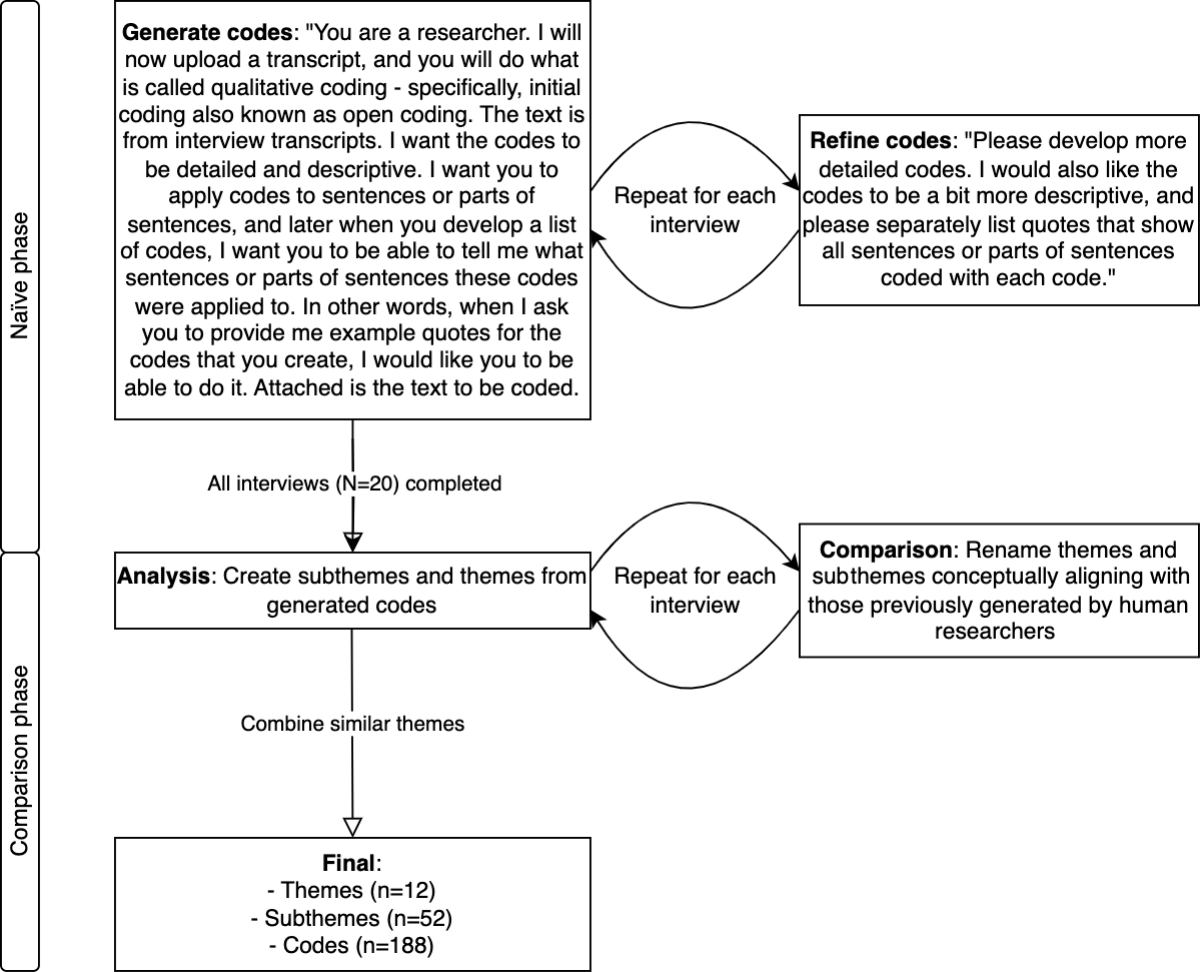
Procedure for using GPT-4 for qualitative description.

Each deidentified transcript underwent text formatting removal before analysis by GPT-4 using a standardized prompt set (Figure 1) [26]. The analysis of the GPT-4–generated output was conducted in 2 phases, the naïve phase and the comparison phase.

In the naïve phase, GPT-4’s outputs for each interview were examined to extract relevant codes and quotes. These were then combined into subthemes, with groupings based on conceptual coherence and content relevance, following a standard qualitative description process [24]. Subsequently, similar subthemes were grouped to form overarching themes. Multiple iterations were conducted to refine the subthemes before synthesizing generalizations that held true across the data. Memo writing was integral to this process, capturing the evolving understanding of the data. Importantly, no discussions with the human-analyst team were conducted during this phase to avoid biasing the process. All interactions and evaluations of GPT-4’s analyses were conducted by a blinded reviewer (KDL) who was not involved in the initial human-driven analysis and kept naïve to its outcomes.

In the comparison phase, AI-identified subthemes and themes were compared against those previously identified through human-driven analysis. This phase focused on identifying parallels and alignments between the 2 analyses to provide a direct comparison.

Interview data were collected in 2021, and human analyses were completed by 2022. All GPT-4 analyses were processed in separate instances on December 1, 2023, using the latest model of GPT-4 available at that time.

### Measures to Ensure Rigor

The analytic team included KDL, who is a medical and data science master’s student, NR, who is a clinical research coordinator with extensive experience in managing and coordinating clinical studies in health care settings, and GMA, who is a fellowship-trained surgeon specializing in urologic conditions, including adult acquired buried penis. In addition, we consulted BNB, an expert in urologic reconstruction who frequently treats patients with buried penis, to provide in-depth clinical insights and ensure the medical accuracy of our interpretations, and RS, a health services researcher and communication scientist with expertise in qualitative methods, to guide us on appropriate methodologies and ensure the rigor of our analyses.

To ensure rigor, we implemented several strategies addressing credibility, transferability, dependability, and confirmability [27]. For credibility, we built patient rapport through prolonged engagement, as most patients had existing longitudinal relationships at the urology clinics where they received care, allowing for deeper insights into their experiences. For transferability, we reported clinical characteristics of the study participants to inform the applicability of our findings to other populations with AABP and used a multi-institutional sampling strategy to account for potential geographic or local institutional characteristics, ensuring broader applicability of our results.

Dependability was ensured through methodological documentation, where all codes, subthemes, and themes were documented at each step to provide transparency and replicability of our coding decisions. We also maintained detailed audit trails of raw outputs from GPT-4, processed outputs, and the subsequent organization into subthemes and themes, which the team reviewed to ensure consistency and reliability. Confirmability was achieved by having BNB, an expert in urologic reconstruction, review the study findings and provide critical insights during the design phase, and RS, who provided qualitative methodological support. In addition, data were shared with the entire research team, and feedback from all coauthors was incorporated into subsequent interpretation and analysis.

### Comparison of Analyses

Qualitative analyses, including themes and subthemes, were summarized using descriptive statistics, including frequencies and proportions. To visually represent an agreement between human and AI-identified themes (validity), an agreement matrix was constructed. We measured interrater reliability using Cohen κ coefficient. A separate analysis was performed 10 times on the same interview transcript to assess the reliability of GPT-4’s analysis. Themes identified exclusively by GPT-4 were highlighted with exemplar quotes that best represented each theme. All analyses were performed using R statistical software (version 4.3.1; The R Foundation).

### Ethical Considerations

The study was approved by the University of California San Francisco (UCSF) institutional review board (IRB; 20-32062), and consent was obtained from all participants. In addition to the original study’s IRB approval, we obtained an exemption from our institution’s IRB for the secondary analysis using GPT-4, as the data were deidentified. Before analysis, all transcripts were reviewed to ensure that they contained no protected health information or identifiable data to maintain participant confidentiality. We used a private instance of GPT-4, known as Versa, which operates independently of OpenAI’s commercial model and does not retain or learn from the data inputted [28]. This instance was used to develop our AI qualitative analysis methodology. For subsequent analyses, all data were confirmed to be thoroughly deidentified before using the commercial version of GPT-4.

## Results

### Study Population

Participant characteristics are summarized in Table 1. Participants’ mean age and BMI were 58.8 (SD 13.9) years and 41.1 (SD 9.4) kg/m^2^, respectively. Most participants were White (17/20, 85%), married (12/20, 60%), heterosexual (19/20, 95%) men residing in the Western region of the United States (10/20, 50%). In total, 55% (11/20) of participants underwent surgical correction of their AABP, with interviews conducted at an average of 497 (SD 666) days after surgery.

**Table 1:** Participant demographics and characteristics.

Characteristics		Values
Age (years), mean (SD)		58.8 (13.9)
BMI (kg/m^2^), mean (SD)		41.1 (9.4)
**Self-identified race, n (%)**		
	White	17 (85)
	Black or African American	1 (5)
	Other	2 (10)
	Hispanic or Latin ethnicity	3 (15)
**Relationship status, n (%)**		
	Married	12 (60)
	Single	6 (30)
	In a relationship	2 (10)
**Sexual orientation, n (%)**		
	Heterosexual	19 (95)
	Homosexual	1 (5)
**Region, n (%)**		
	West	10 (50)
	Northeast	7 (35)
	Midwest	2 (10)
	South	1 (5)
**Patients who underwent AABP^a^ surgical correction (n=11, 55%), n (%)**		
	Escutcheonectomy	9 (45)
	Excision of penile skin with split-thickness skin graft	6 (30)
	Ventral slit scrotal flap	5 (25)

^a^AABP: adult-acquired buried penis.

### Qualitative Description

Table 2 presents a comparative analysis of themes and subthemes identified by human researchers versus GPT-4. “Urinary issues” were common in interviews analyzed by human researchers (19/20, 95%) and GPT-4 (15/20, 75%). Issues with “spray or stream” were a notable subtheme (12/20, 60% humans vs 7/20, 35% GPT-4). “Sexual issues” were prominently coded as well, present in 95% (19/20) of human-analyzed interviews and 80% (18/20) by GPT-4, with “inability to perform intercourse” coded as a subtheme more frequently by human researchers (12/20, 60% vs 6/20, 30%). Humans coded a broader array of sexual function issues, such as “pain with sex or masturbation” (7/20, 35%) and “difficulty with sex or masturbation” (4/20, 20%). “Mental health issues” were similarly recognized by both humans and GPT-4 (11/20, 55%, both), with “depression” more frequently coded by humans compared with GPT-4 (10/20, 50% vs 4/20, 20%, respectively). “Impact on social life” was an additional significant theme, with humans coding “issues using public restrooms” (12/20, 60%), while GPT-4 emphasized “struggles with romantic relationships” (9/20, 45%). Both methods identified “hygiene issues” (14/20, 70% humans vs 13/20, 65% GPT-4), highlighting difficulties in maintaining cleanliness. Human researchers uniquely identified “contributing factors” as a theme in all interviews.

**Table 2:** Human researchers versus GPT-4 qualitative analysis.

Themes and subthemes		Human researchers, n (%)	GPT-4, n (%)
**Urinary issues**		19 (95)	15 (75)
	Spray or stream	12 (60)	7 (35)
	Hovers over toilet	8 (40)	—^a^
	Pain with urination	7 (35)	3 (15)
	History of urethral stricture disease	3 (15)	—
	Incontinence	3 (15)	3 (15)
	Incomplete bladder emptying	3 (15)	2 (10)
	Sits to urinate	2 (10)	—
	Smelly urine	1 (5)	1 (5)
	Trouble with catheter	1 (5)	—
	Uses shower or tub to urinate	1 (5)	—
	Frequent urination	—	2 (10)
	Getting up at night to urinate	—	1 (5)
**Sex issues**		19 (95)	16 (80)
	Unable to perform intercourse	12 (60)	6 (30)
	Unable to get erection	9 (45)	3 (15)
	Pain with sex or masturbation	7 (35)	—
	Difficulty with sex or masturbation	4 (20)	—
	Painful erection	4 (20)	—
	Unable to maintain erection	3 (15)	—
	Avoids sex	2 (10)	4 (20)
	Unable to orgasm	2 (10)	—
	Reduced genital sensation	1 (5)	—
	Takes longer to orgasm	1 (5)	—
	Pain with ejaculation	1 (5)	—
	Intercourse not enjoyable	1 (5)	—
	Adaptive masturbation techniques	—	2 (10)
	Poor cosmetic appearance	—	2 (10)
	Painful erection	—	2 (10)
	Brittle skin	—	1 (5)
	Unable to use condom	—	1 (5)
	Overuse of pornography	—	1 (5)
**Mental health issues**		11 (55)	11 (55)
	Depression	10 (50)	6 (30)
	Feels like less of a man	7 (35)	4 (20)
	Anxiety	4 (20)	2 (10)
	Decreased self-esteem	3 (15)	—
	Stress	1 (5)	1 (5)
	Emotional turmoil	—	2 (10)
	Loss of confidence	—	1 (5)
	Guilt	—	1 (5)
**Impacts social life**		16 (80)	15 (75)
	Issues using public restrooms	12 (60)	8 (40)
	Avoids travel	6 (30)	—
	Struggles with romantic relationships	—	9 (45)
	Mobility impairment	—	6 (30)
	Spousal support	—	3 (15)
	Avoids hobbies	—	1 (5)
	Avoids social activities	—	1 (5)
	Negative impact on career	—	1 (5)
**Hygiene issues**		14 (70)	13 (65)
	Hard or effort to clean	11 (55)	11 (55)
	Skin tearing	7 (35)	—
	Penile bleeding	6 (30)	2 (10)
	Infections	—	6 (30)
**Contributing factors**		20 (100)	—
	Worse after weight gain	14 (70)	—
	Worse after multiple surgeries	8 (40)	—
	Worse after weight loss	4 (20)	—
	Improvement after weight loss	0 (0)	—

^a^Not applicable.

### Validity and Reliability of GPT-4 Analysis

**Figure 2:**
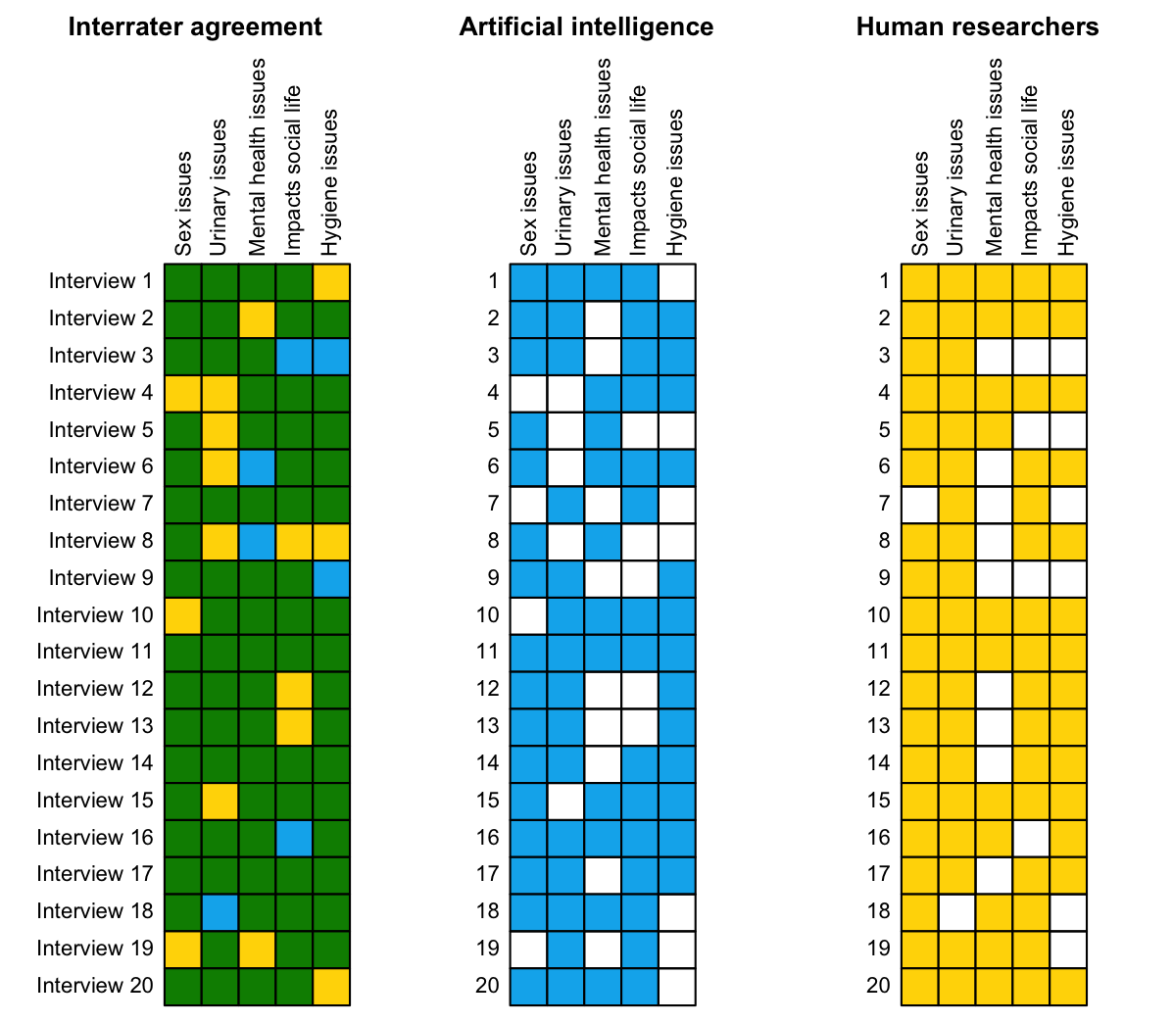
Themes identified per interview by GPT-4 versus human researchers.

To further assess the validity of GPT-4 analysis, we generated an agreement matrix comparing themes coded by human researchers and GPT-4 per interview (Figure 2). There were 63 instances where both human and GPT-4 analyses agreed on the presence of a theme, and 14 instances of agreement on a theme being absent. There was disagreement in 23 cases—16 where humans identified a theme that GPT-4 did not and 7 where GPT-4 identified a theme that humans did not (Table 3). The overall Cohen κ coefficient was 0.401, indicating moderate agreement. Boxes depict interview theme analysis. The blue (AI) and yellow (humans) squares indicate presence and green squares reflect agreement on presence or absence.

We assessed reliability by analyzing the same interview transcript 10 times with the same prompt set (Table 4). There was consistent identification of “body image struggles or disfigurement” and “chronic pain and discomfort,” both appearing in all iterations (10/10, 100%). “Depression” was also frequently coded, appearing in 90% (9/10) of analyses. High reliability was observed for “motivated to have surgery,” “uses shower or tub to urinate,” and “weight challenges,” each occurring in 80% (8/10) of the analyses. Other codes such as “issues using public restrooms,” “unable to perform intercourse,” and “negative health care experiences” were present in 70% (7/10) of iterations. Codes for “hard or effort to clean,” “decreased self-esteem,” and “necrotizing fasciitis diagnosis” were identified 60% (6/10) of the time. Codes were less frequent for “urinary tract infections” (3/10, 30%), “sits to urinate” (2/10, 20%), and a cluster of codes that included “dependency on others for care,” “social isolation and loneliness,” “high frequency of urination,” “anxiety,” “loss of physical autonomy,” “financial burden,” and “hematuria,” each appearing once (1/10, 10%).

**Table 3:** Codes and exemplar quotes identified exclusively by GPT-4.

Interview number	GPT-4 code applied: exemplar quote	Theme
3	Impact on marital relationship: “I am married? And you know it’s it is... strained or? I wasn’t meeting her needs.”	Impacts social life
3	Hygiene management efforts: “I try to keep myself pretty clean... I really tried to wash my genitals really well.”	Hygiene issues
6	Mental health impact and resilience: “Yes in some ways it did affect me but other ways I don’t really don’t think it did.”	Mental health issues
8	Mental health and self-image concerns: “the preconceived notion you know but the man’s function is supposed to be.”	Mental health issues
9	Improved hygiene post surgery: “I actually feel that hygiene became a lot easier simply because I didn’t have to dig my finger in and run around the shaft to try and wash everything out.”	Hygiene issues
16	Day-to-day discontent and social withdrawal: “It’s just I just I would hate for other candidates that going forward thinking there is nothing that can be done need to be here they need to have options on the table.”	Impacts social life
18	Urinary dysfunction and social anxiety: “I would say they’re abnormal for somebody my age a lot of times it’s needing the needing to push… And that can cause anxiety in a public sort of restroom atmosphere.”	Urinary issues

**Table 4:** Reliability of GPT-4–generated codes.

Code^a^	Participants, n (%)
Body image struggles or disfigurement	10 (100)
Chronic pain and discomfort	10 (100)
Depression	9 (90)
Motivated to have surgery	8 (80)
Uses shower or tub to urinate	8 (80)
Weight challenges	8 (80)
Issues using public restrooms	7 (70)
Unable to perform intercourse	7 (70)
Negative health care experiences	7 (70)
Hard or effort to clean	6 (60)
Decreased self-esteem	6 (60)
Necrotizing fasciitis diagnosis	6 (60)
Urinary tract infections	3 (30)
Sits to urinate	2 (20)
Dependency on others for care	1 (10)
Social isolation and loneliness	1 (10)
High frequency of urination	1 (10)
Anxiety	1 (10)
Loss of physical autonomy	1 (10)
Financial burden	1 (10)
Hematuria	1 (10)

^a^Presence of codes from the same interview analyzed 10 times by GPT-4. Each code was counted only once per analysis, indicating whether it was identified (present) or not (absent) during each separate analysis.

## Discussion

### Principal Results

In this investigation, we directly compared the performance of AI (GPT-4) with human researchers in conducting a qualitative analysis of interviews with patients affected by AABP. Our study is the first of its kind, to our knowledge, to perform such a direct comparison, highlighting the potential use of AI in qualitative research. By using generalized prompts, our method allows researchers without specialized NLP knowledge to use GPT-4 for rigorous qualitative analysis, significantly reducing the time investment required.

Our results showed moderate alignment between GPT-4 and human analyses in identifying key themes, including urinary challenges, sexual health issues, and mental health impacts. Human analysis identified more subthemes, capturing the data’s complexities more thoroughly than GPT-4. This difference may stem from GPT-4’s token size limitations, which restrict its ability to perform comprehensive analyses as the input length increases [29]. The reliability tests revealed that while GPT-4 consistently recognized key codes, its identification of subtler codes was more variable. This suggests that implementing repeated analysis cycles, similar to the human multirater approach, could refine AI’s analytical reliability. Overall, our findings underscore a complementary role for AI and human collaboration in qualitative research, where each can augment the strengths of the other.

The question of how to evaluate the accuracy and reliability of AI-driven analysis is crucial for future research. We adopted a quantitative approach to directly compare the presence of themes and subthemes in both human and AI analyses. By calculating Cohen κ, a statistic that measures interrater reliability by considering the agreement occurring by chance, we provided an objective assessment of the consistency of themes identified by GPT-4 compared with human analysis, presupposing human analysis as the “gold standard.” In addition, to ensure consistency in GPT-4’s outputs, we conducted multiple iterations of the same interview transcript analysis, analogous to traditional qualitative research methods where multiple analysts and iterative coding processes are used to standardize analyses and minimize biases. It is important to note that while these quantitative metrics offer a clear criterion for comparison, they may not fully capture the depth and richness of qualitative insights. GPT-4 has demonstrated the ability to detect subtle nuances and emotional contexts from text data, suggesting that incorporating more qualitative approaches in AI analysis evaluation could enhance the understanding of its analytical capabilities [30,31].

### Limitations

A primary limitation of this study arises from the comparison phase, where themes and subthemes generated by GPT-4 were aligned with those identified by human researchers. Although a blinded reviewer was used to mitigate potential bias, the subjective nature of qualitative analysis means that a degree of bias is likely to remain. This is a common challenge in qualitative research, where analysts’ subjective interpretations inherently influence their analysis. However, it can be argued that the use of a large language model such as GPT-4 may present a more objective method of analysis compared with the potential variability inherent between different human researchers’ analyses, due to the large language model’s consistent application of its transformer model.

We deliberately chose qualitative description as our analytic approach, favoring the accuracy to source material over depth of analysis. Qualitative description involves the systematic categorization and interpretation of qualitative data to uncover patterns and insights while staying close to the original data [22-24]. A more context-based approach, such as thematic analysis, could generate richer themes and subthemes but poses challenges for comparability. More interpretative methods may introduce subjectivity, reducing reproducibility. While our methodological choice ensures that our study remains accessible as a framework for others to build on and develop more interpretative techniques, the need for comparison limited our depth of insights.

Qualitative methods have inherent limitations, such as potential bias and limited generalizability due to smaller, nonrandom samples, and aim to produce in-depth insights and understanding rather than population inferences [32,33]. Consequently, our findings may not capture the full diversity of patient experiences, potentially limiting the generalizability of our results. Nevertheless, our study primarily aims to provide a comparative analysis, focusing on GPT-4 as a suitable tool for qualitative research applications.

As GPT-4 and other large language models advance, their analytical capabilities are expected to become more sophisticated, which may alter their proficiency in qualitative analysis. For example, while GPT-3.5 scored in the bottom 10% on a simulated bar examination, GPT-4 has demonstrated a significant improvement, placing within the top 10% of test takers [11]. The study’s findings are therefore a snapshot of GPT-4’s capabilities at a specific point in time and may not fully represent its future potential in qualitative analysis. Despite this limitation, the current trajectory of AI indicates that the use of GPT-4 and similar large language models in qualitative research is likely to become increasingly robust and refined.

### Comparison With Previous Work

While studies applying GPT-4 or other large language models to qualitative research are limited, a growing body of work has compared the performance of OpenAI’s GPT models, including GPT-3, -3.5, and -4, with that of humans in academic research and medical education [12-15]. Wang et al [34] found that while ChatGPT can generate accurate and relevant information, it is not without gaps when compared with official sources, indicating a need for supplementary validation from reliable references. Other studies have shown that ChatGPT can mimic the style of human-written research abstracts, albeit with limitations in quality and accuracy, as indicated by the ability of blinded reviewers to distinguish AI-generated content [35]. In the field of medical education, ChatGPT has been shown to outperform medical students on examinations, suggesting valuable applications in examination preparation [36]. Similarly, ChatGPT’s performance on the United States Medical Licensing Examination (USMLE) further showcases the potential use of AI in medical education, where it achieved scores near the passing threshold without specialized training [37]. These findings emphasize that while advanced large language models such as GPT-4 are becoming increasingly competent in complex tasks, their current role remains complementary to human expertise.

The application of GPT-4 and other large language models to health care is a burgeoning field with substantial promise, resting on the fundamental ability of AI to process qualitative data efficiently. In patient care, large language models can enhance communication by translating complex medical language into more accessible terms for health care providers and patients [38]. The performance of large language models on medical licensing examinations also indicates their potential use in supporting clinical decision-making [39]. In administrative contexts, large language models are particularly valuable for generating concise clinical summaries and synthesizing extensive electronic medical record documentation; tasks that typically consume considerable time for health care professionals. The integration of large language models into administrative workflows may increase efficiency and allow clinicians to allocate more time to direct patient care. Health care companies are already beginning to integrate large language models into electronic health records, such as Epic’s recent partnership with Microsoft to embed Azure OpenAI service into its own electronic health record systems [40].

Despite its promise, the integration of large language models in health care raises several ethical concerns that warrant careful consideration [41]. Foremost among these is data privacy, particularly regarding the handling of sensitive patient information, necessitating robust safeguards against data breaches. The opacity of these models, due to the unavailability of public training data sets and model weights, poses another concern as it obscures the understanding of their decision-making processes and challenges their trustworthiness in clinical applications [42]. In addition, the commercialization of large language models by major corporations, such as OpenAI, Microsoft, Meta, and Google, brings into question the potential influence of commercial interests on model development and deployment, possibly overshadowing patient welfare. A crucial concern is the risk of patient harm arising from incorrect or biased models, emphasizing the need for rigorous testing and validation of large language models to ensure their reliability and prevent adverse clinical outcomes [43].

### Conclusions

Our research demonstrates that large language models like GPT-4 can discern key themes from qualitative health care data when used with standardized prompts. This “out of the box” approach aligns moderately well with qualitative description analysis by human analysts. Future work should use more context-based prompts for deeper and richer themes. As this may introduce greater subjectivity, researchers should also explore iterative analyses, such as synthesizing output from multiple iterations, to improve large language model output reliability. In addition, researchers should assess the qualitative analytic abilities of other popular models like Gemini (Google), Llama (Meta), and Claude (Anthropic AI), and develop methods to circumvent the token limitations inherent in models such as GPT-4 by segmenting qualitative data inputs, enriching the depth and breadth of qualitative analyses.

## Abbreviations

AABP: adult-acquired buried penis
AI: artificial intelligence
BERT: Bidirectional Encoder Representations from Transformers
IRB: Institutional Review Board
NLP: natural language processing
TURNS: Trauma and Urologic Reconstructive Network of Surgeons
UCSF: University of California San Francisco
USMLE: United States Medical Licensing Examination
